# Cleft palate: a rare complication following esophageal stent implantation

**DOI:** 10.1055/a-2067-4681

**Published:** 2023-04-21

**Authors:** Wei Liu, Ou Chen, Lifan Zhang, Bing Hu

**Affiliations:** 1 Department of Gastroenterology and Hepatology, West China Hospital, Sichuan University, Chengdu, China; 2 Department of Gastroenterology, Ya’an People’s Hospital, Ya’an, China

A 50-year-old woman was referred to our hospital because of increasing dysphagia. She had been diagnosed with systemic lupus erythematosus 2 years previously and started on treatment with tacrolimus and corticosteroids. Multiple diverticula of the esophagus were confirmed and the largest diverticulum was located next to the cardia. Esophagogram showed delayed passage of contrast into the stomach, and gastroscopy revealed a dilated esophagus with retained liquid and increased resistance at the cardia ( Fig. 1 a–c ). The patient refused to undergo endoscopic or surgical myotomy as she was concerned about the treatment risks. Therefore, esophageal stent implantation was believed to be an option for this case.

**Fig. 1:**
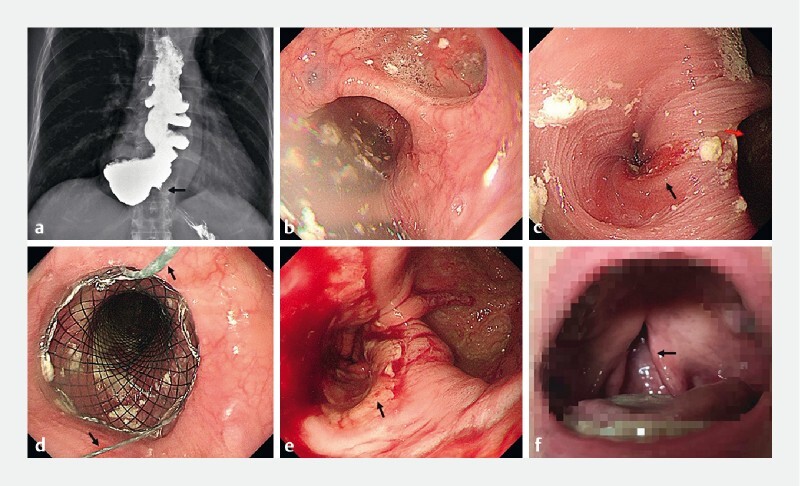
Cleft palate: a rare complication following esophageal stent implantation. **a** Esophagogram showed multiple diverticula of the esophagus and delayed passage of contrast into the stomach (black arrow). **b, c** Gastroscopy revealed multiple diverticula in the dilated esophagus with retained liquid and increased resistance at the cardia (black arrow); the largest diverticulum was located next to the cardia (red arrow). **d** A fully covered retrievable self-expandable metal stent (SEMS) was positioned and deployed, and a thread was fixed to the patientʼs nose to prevent stent migration (black arrow). **e** Stent retrieval was performed 2 months later and endoscopy revealed decreased resistance at the cardia (black arrow). **f** A cleft palate was caused by the thread that fixed the SEMS (black arrow).

During treatment, the most appropriate dimension of the self-expandable metal stent (SEMS) was determined by judging the location of the multiple diverticula based on endoscopic assessment; a fully covered retrievable SEMS (Micro-Tech, Nanjing, China) with a size of 20 mm × 140 mm was selected. The SEMS delivery system was advanced across the cardia over the guidewire, and the SEMS was then positioned and deployed adequately under endoscopic view; a thread was fixed to the patient’s nose to prevent stent migration ( Fig. 1 d ).

Follow-up endoscopy and stent retrieval were performed 2 months later. The dysphagia had improved significantly and endoscopy revealed decreased resistance at the cardia ( Fig. 1 e ). However, the thread that fixed the SEMS had caused a cleft palate ( Fig. 1 f , Video 1 ). After consulting an oral surgeon, repair surgery was not recommended as the patient had no obvious symptoms associated with the cleft palate.

**Video 1** Cleft palate: a rare complication following esophageal stent implantation.


Cleft palate caused by esophageal stent implantation has never been reported before. Although SEMS was effective for this patient, fixing the thread to the nose is very risky and dangerous, and alternative tools (e. g. hemoclips, suturing, over-the-scope clip) should be used to prevent migration and the occurrence of such complications 1 2 3 4 .

Endoscopy_UCTN_Code_CPL_1AH_2AD

## Notes

Endoscopy E-Videos

https://eref.thieme.de/e-videos
*E-Videos* is an open access online section of the journal *Endoscopy* , reporting on interesting cases and new techniques in gastroenterological endoscopy. All papers include a high-quality video and are published with a Creative Commons CC-BY license. Endoscopy E-Videos qualify for HINARI discounts and waivers and eligibility is automatically checked during the submission process. We grant 100% waivers to articles whose corresponding authors are based in Group A countries and 50% waivers to those who are based in Group B countries as classified by Research4Life (see: https://www.research4life.org/access/eligibility/ ). This section has its own submission website at https://mc.manuscriptcentral.com/e-videos

## References

[1] Singla V Khare S Arora A Use of loop and clips to prevent migration of esophageal stent Endoscopy 2021 53 E421 E422 33506470 10.1055/a-1326-1143

[2] Singla V Arora A Khare S A novel technique to prevent migration of esophageal stent Endoscopy 2020 52 1040 1041 32303085 10.1055/a-1149-1084

[3] Shah E D Hosmer A E Patel A Valuing innovative endoscopic techniques: endoscopic suturing to prevent stent migration for benign esophageal disease Gastrointest Endosc 2020 91 278 285 31449789 10.1016/j.gie.2019.08.020

[4] Watanabe K Hikichi T Nakamura J Feasibility of esophageal stent fixation with an over-the-scope-clip for malignant esophageal strictures to prevent migration Endosc Int Open 2017 5 E1044 E1049 29090243 10.1055/s-0043-111793 PMC5658212

